# Seed-Specific Overexpression of the Pyruvate Transporter *BASS2* Increases Oil Content in *Arabidopsis* Seeds

**DOI:** 10.3389/fpls.2017.00194

**Published:** 2017-02-20

**Authors:** Eun-Jung Lee, Minwoo Oh, Jae-Ung Hwang, Yonghua Li-Beisson, Ikuo Nishida, Youngsook Lee

**Affiliations:** ^1^Division of Integrative Biosciences and Biotechnology, Plant Cell Biology Laboratory, Pohang University of Science and TechnologyPohang, South Korea; ^2^Department of Plant Biology and Environmental Microbiology, CEA/Centre National de la Recherche Scientifique/Aix-Marseille University, CEA CadaracheMarseille, France; ^3^Division of Life Science, Graduate School of Science and Engineering, Saitama UniversitySaitama, Japan

**Keywords:** seed oil yield, pyruvate transporter, *BASS2*, seed-specific promoter, bioenergy

## Abstract

Seed oil is important not only for human and animal nutrition, but also for various industrial applications. Numerous genetic engineering strategies have been attempted to increase the oil content per seed, but few of these strategies have involved manipulating the transporters. Pyruvate is a major source of carbon for *de novo* fatty acid biosynthesis in plastids, and the embryo's demand for pyruvate is reported to increase during active oil accumulation. In this study, we tested our hypothesis that oil biosynthesis could be boosted by increasing pyruvate flux into plastids. We expressed the known plastid-localized pyruvate transporter *BILE ACID:SODIUM SYMPORTER FAMILY PROTEIN 2* (*BASS2*) under the control of a seed-specific soybean (*Glycine max*) *glycinin-1* promoter in *Arabidopsis thaliana*. The resultant transgenic *Arabidopsis* plants (OEs), which expressed high levels of *BASS2*, produced seeds that were larger and heavier and contained 10–37% more oil than those of the wild type (WT), but were comparable to the WT seeds in terms of protein and carbohydrate contents. The total seed number did not differ significantly between the WT and OEs. Therefore, oil yield per plant was increased by 24–43% in the OE lines compared to WT. Taken together, our results demonstrate that seed-specific overexpression of the pyruvate transporter *BASS2* promotes oil production in *Arabidopsis* seeds. Thus, manipulating the level of specific transporters is a feasible approach for increasing the seed oil content.

## Introduction

Seed oil is an important source of energy, and is in increasing demand for various industrial applications (Dyer et al., 2008; Hayden et al., 2011). Thus, methods to increase seed oil yield are being actively investigated. Many efforts to boost seed oil yield have involved genetically engineering *Arabidopsis* and other plants to overexpress transcription factors and enzymes involved in fatty acid biosynthesis and lipid production and storage (Napier et al., 2014). For example, overexpression of the transcription factor *WRINKLED 1* (*WRI1*), which controls the expression of genes involved in lipid metabolism, including glycolysis and fatty acid biosynthesis, increased seed oil content by 10–20% compared to the wild type (Cernac and Benning, 2004; Baud et al., 2007, 2009; Maeo et al., 2009). Furthermore, the seed-specific overexpression of the *Arabidopsis* acyl-CoA:diacylglycerol transferase 1 (*DGAT1*), which catalyzes the formation of triacylglycerol (TAG) from diacylglycerol (DAG) and acyl-CoA, increased oil content by 11–28% compared with the control (Jako et al., 2001).

In addition to manipulating the levels of transcription factors and enzymes involved in lipid biosynthesis, manipulating the expression of transporters, particularly those that compartmentalize precursors in the intracellular compartments where lipid biosynthesis occurs, might be a useful approach for increasing oil content. During seed development, sucrose imported from maternal tissues is converted to glucose 6-phosphate (G6P) (Barratt et al., 2009). Part of the G6P can be transported to the plastid through Glc6P/phosphate translocator (GPT) (Kammerer et al., 1998), and can provide pyruvate through glycolytic reactions in the plastid. Part of the G6P is also metabolized in the cytosol to phosphoenolpyruvate (PEP), some of which enters the plastid via the phosphoenolpyruvate/phosphate translocator (PPT) and the other part of PEP can be converted to pyruvate by cytosolic pyruvate kinase (Andre et al., 2007). Once inside the plastid, PEP is converted into pyruvate by plastidial pyruvate kinase. Plastidial pyruvate can be further metabolized either through the methylerythrol phosphate (MEP) pathway, or is converted by plastidial pyruvate dehydrogenase into acetyl-CoA, which is a substrate of acetyl-CoA carboxylase (ACCase), initiating *de novo* fatty acid biosynthesis (Rawsthorne, 2002). Current evidence suggests that plastidial fatty acid biosynthesis largely depends on the import of cytosolic PEP into the plastid by PPT during seed development of *Arabidopsis thaliana* (Fischer et al., 1997; Knappe et al., 2003). Adequate provision of PEP by the PEP transporter PPT appears to be essential for the biosynthesis of lipids and other storage substances. An *Arabidopsis* mutant defective in both PPT and the plastid-localized enolase (ENO1) involved in glycolytic PEP provision exhibits retarded vegetative growth and defective flower development (Kubis et al., 2004). Moreover, this double mutant exhibited frequent seed abortion and diminished oil amount in seeds, caused by disruption of multiple pathways including fatty acid synthesis.

Another transporter potentially important for seed oil accumulation is the pyruvate transporter at the plastid envelope. The uptake of pyruvate into plastids seems to be an important step in fatty acid biosynthesis during seed development, because pyruvate uptake into plastids increases during embryo development, and isolated plastids from oilseed rape embryos are able to use pyruvate as a substrate for fatty acid biosynthesis (Eastmond and Rawsthorne, 2000). A pyruvate transporter named *BILE ACID:SODIUM SYMPORTER FAMILY PROTEIN 2 (BASS2)* (Furumoto et al., 2011) has recently been identified and shown to localize to the plastid membranes of leaves. BASS2 is a sodium-dependent pyruvate transporter functioning in C4 photosynthesis and in the MEP pathway in C3 plants (Furumoto, 2016). Recently, it was reported that a putative pyruvate transporter *TaBASS2* isolated from wheat enhanced salinity tolerance when transgenically expressed in wheat and Arabidopsis (Zhao et al., 2016). Moreover, BASS2 and its homolog might function in oil seed plastids, since *BASS2* is expressed during the early stages of seed development (Arabidopsis eFP browser, http://bar.utoronto.ca/efp/cgi-bin/efpWeb.cgi), albeit at a much lower level than in the leaves, and a homolog of *AtBASS2* was found to be expressed at 4.7 times higher levels in mesocarp of oil palm (*Elaeis guineensis Jacq*) than in that of date palm (*Phoenix dactylifera*) (Bourgis et al., 2011).

**Figure 1:**
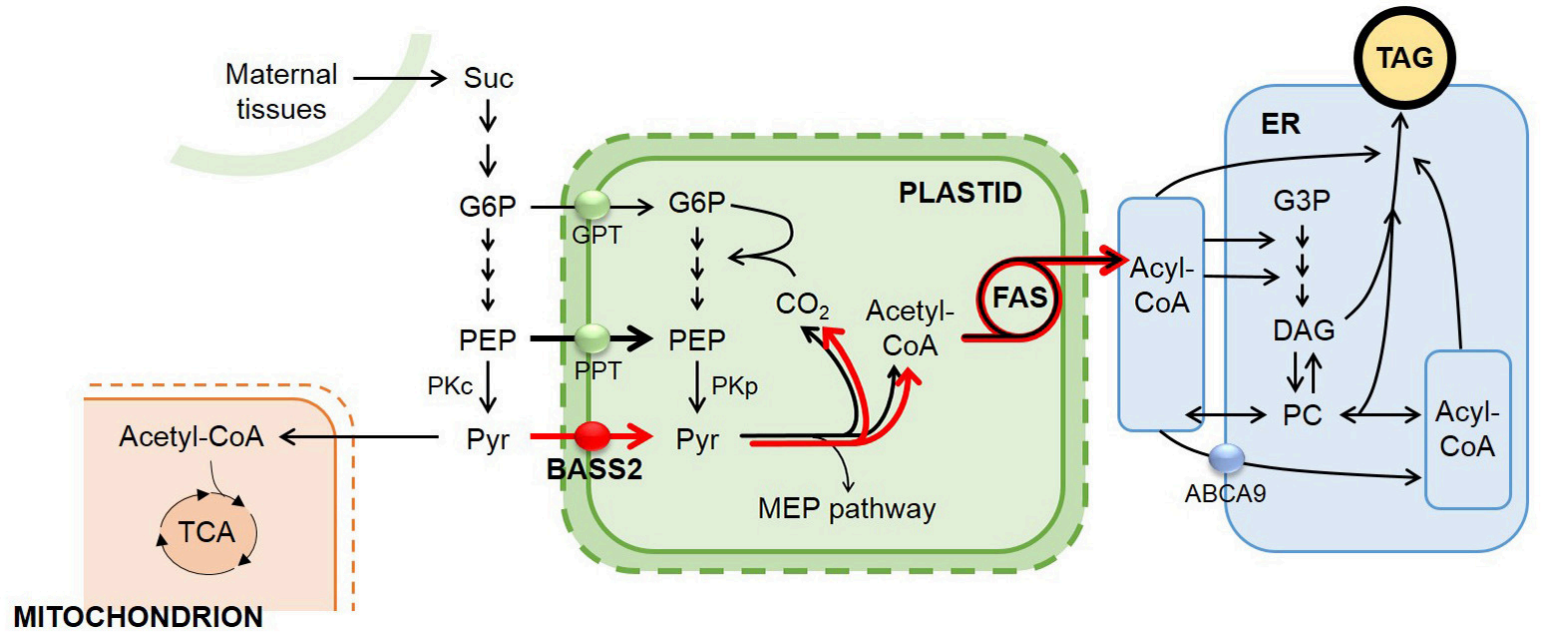
**Our strategy to increase seed oil accumulation by overexpressing the plastidial pyruvate transporter BASS2 under the control of a seed-specific promoter in developing ***A. thaliana*** seeds**. A simplified diagram depicting the oil metabolism pathway in developing *A. thaliana* seeds. During seed development, sucrose imported from maternal tissues is converted to G6P. Then, G6P is metabolized to PEP. In the cytosol, PEP is either converted to pyruvate or enters a plastid via PPT. In plastids, pyruvate is used as a substrate for fatty acid biosynthesis, and the resulting fatty acids are channeled into the TAG biosynthesis pathway in the ER. Cytosolic pyruvate is used for many metabolic pathways, such as protein biosynthesis and the TCA cycle. By increasing pyruvate flux into plastids by the seed-specific overexpression of the pyruvate transporter BASS2, we expected to increase fatty acid biosynthesis in the plastids and finally enhance TAG biosynthesis in developing *Arabidopsis* seeds. This figure was modified from van Erp et al. (2014) Plant Physiology. Ovals indicate transporters responsible for each transport process: GPT, Glucose-6-phosphate translocator (green); PPT, phosphoenolpyruvate/phosphate translocator (green); BASS2, bile-acid sodium symporter 2 (red); ABCA9; ATP-binding cassette A subfamily member 9 (blue). Other abbreviations are: Suc, sucrose; G3P, Glucose-3-phosphate; G6P, Glucose-6-phosphate; PEP, phosphoenolpyruvate; PKc, cytosolic pyruvate kinase; PKp, plastidic pyruvate kinase; Pyr, pyruvate; FAS, fatty acid synthase complex; MEP, methylerythritol phosphate; PC, phosphatidylcholine; DAG, diacylglycerol; TAG, triacylglycerol; Acyl-CoA, acyl-Coenzyme A; Acetyl-CoA, acetyl-Coenzyme A; TCA, tricarboxylic acid. Black lines indicate pathways active in the cell during seed maturation. Red indicates the flux of newly incorporated pyruvate by pyruvate transporter (BASS2) in transgenic lines.

In this study, we tested our hypothesis that increased pyruvate uptake into the plastids of developing seeds by overexpressing the pyruvate transporter *BASS2* would increase the supply of carbon precursors, thus facilitating *de novo* fatty acid biosynthesis and eventually enhancing seed oil production (Figure 1). To test our hypothesis, we generated transgenic *Arabidopsis* plants that overexpressed *BASS2* under the control of a seed-specific promoter from soybean (*Glycine max*). We report that the seed-specific *BASS2*-overexpressing *Arabidopsis* plants produced seeds with an 8–27% increase in oil content.

## Materials and methods

### Plant materials and growth conditions

Wild-type and transgenic Arabidopsis (*Arabidopsis thaliana*) plants were of the Col-0 ecotype. Seeds were sterilized and imbibed in darkness for 3 days at 4°C. Seeds were sown on half-strength MS medium (Murashige and Skoog, 1962), pH 5.8, containing 1% sucrose and 0.8% agar, and placed in a growth chamber set to a light/dark period of 16 h (22°C)/8 h (18°C) and photon flux density of 40 μmol m^−2^ s^−1^ light. After 2 weeks, seedlings were transferred to soil and grown in either a growth chamber or greenhouse.

### Construct preparation and *Arabidopsis* transformation

*BASS2* (At2g26900) was amplified by PCR from Arabidopsis complementary DNA using the following primer pair: 5′-GAATTCATGGCTTCCATTTCCAGAATCT-3′ and 5′-CTCGAGTTACTCTTTGAAGTCATCCTTG-3′. The product was cloned into T-blunt vector using the T-blunt™ PCR Cloning Kit (Solgent). *BASS2* CDS was excised using *Eco*RI and *Xho*I and inserted into the pBinGlyBar1 vector using the T4 DNA Ligase Kit (Invitrogen) (Nguyen et al., 2013). The construct was transformed into *Agrobacterium tumefaciens* strain GV3101 by electroporation and into Arabidopsis by the floral dip method (Clough and Bent, 1998). Transformants containing *ProGly:BASS2* were selected by growing the plants in medium containing the pesticide BASTA at a final concentration of 15 μg/ml.

### Transcript analysis

The developing siliques from 12 to 14 DAF T2 plants in seed-specific *BASS2*-overexpressing lines and WT were sampled and frozen in liquid nitrogen. RNA was extracted by phenol-chloroform method and treated with DNase I for 30 min to digest contaminating DNA in samples. The biosynthesis of complementary DNA was carried out using GoScript™ reverse transcriptase from Promega. Real-time PCR was performed using SYBR green. PCR conditions were as follows: 94°C for 3 min, 45 cycles of 94°C for 5 s, 56°C for 15 s, and 72°C for 30 s, and one cycle of 94°C for 15 s, 60°C for 30 s, and 95°C for 15 s. The expression of the housekeeping gene *UBQ11* (At4g05050) was used as a reference. The expression level was normalized by that of UBQ11. The primer pairs used for real-time PCR were (*BASS2*: 5′-AGGTGACTTACCTGAGAGTACT-3′ and 5′-GTAAGTAGCAACGTTTGACGC-3′) and (*UBQ11*: 5′-GAACCAAGTTCATGTATCGT-3′ and 5′-ACACTCATCAAACTAAGCAC-3′).

### Seed size measurement

Seeds were observed using a dissecting microscope and photographs of seeds were taken under the same magnification. Seed size was measured using Image J software, and normalized to that of the WT.

### Metabolite analyses

#### Fatty acid composition and oil content

Seeds were placed in a glass tube with a Teflon screw cap and 10 μl of 2 mM triheptadecanoin (C17:0 TAG) was added as an internal standard for quantification. Then, 1 ml of 5% (v/v) H_2_SO_4_ in methanol with 300 μl toluene as a co-solvent was added to the glass tube. Samples were incubated for 90 min at 90°C to convert oils into their FAMEs. FAMEs were extracted with hexane and 1.5 ml of 0.9% KCl was added to enhance phase separation. FAMEs were quantified using gas chromatography-mass spectrometry (GC-MS) on a HP-INNOWAX capillary column (30 m, 0.25 mm, 0.25 μm) with SHIMADZU GC-2010. The TAG content was estimated based on the content of eicosenoic acid (C20:1), a signature fatty acid of crucifer seed oils. Fatty acid composition was expressed in mol%.

#### Protein extraction and quantification

Protein was extracted from 50 seeds using extraction buffer (100 μl of 1% SDS and 6 M urea) after grinding. Samples were centrifuged at 10,000 g for 10 min. The supernatant was used for protein quantification using the Bio-Rad Protein Assay Kit (Bio-Rad, USA). A known amount (0, 2, 4, 8, 16, and 32 μg) of BSA was used to generate a standard curve. After adding Bio-Rad Protein Assay solution to BSA standard and samples, OD_595nm_ was measured using a spectrophotometer (Pharmaspec UV-1700, Shimadzu).

#### Sucrose and starch extraction and quantification

Carbohydrates were analyzed as previously described (Focks and Benning, 1998) with some modifications. Three hundred seeds were homogenized in 80% (v/v) ethanol and incubated at 70°C for 90 min. After centrifugation at full speed for 5 min at room temperature, the supernatant was transferred to a new test tube. The pellet was extracted three times with 400 μl of 80% (v/v) ethanol, and the solvent of the combined supernatants was evaporated under a vacuum freeze dryer. This residue was dissolved in 100 μl of water and used for sucrose quantification. The insoluble fraction from the ethanol extraction was suspended in 200 μl of 0.5 M KOH and incubated at 95°C for 30 min. After the addition of 100 μl of 1 M acetic acid and centrifugation for 5 min at full speed, the supernatant was used for starch quantification. Sucrose and starch contents were determined using Sucrose colorimetric/fluorometric assay kit and Starch colorimetric/fluorometric assay kit from BioVision (Bio Vision, USA).

#### Seed yield measurement

Four individual plants for each seed-specific *BASS2*-overexpressing line and the WT were harvested to measure the total seed yield of plants grown in the greenhouse. To count silique number per plant and seed number per silique, plants with inflorescence meristems on the main stem that had ceased growing were used. Then, the number of siliques on the main stem was counted. Developing siliques were sampled and treated in 1:1 (v/v) acetic acid/ethanol solution for 3 h. The samples were incubated in 1 N NaOH solution overnight and transferred to 50% glycerol solution. The total seed number of plants was estimated by multiplying the silique number per plant and the seed number per silique.

#### Seed germination and seedling growth assays

The seeds were imbibed in water for 1 h and were then sown in plates containing half-strength MS-agar medium. Other growth conditions were the same as described above. The number of germinated seeds, and the number of roots that reached a line drawn 2 cm below the seeds were counted every 12 h until 10 d after sowing. The germination results were plotted to obtain germination curves. The time required for 50% of the seeds to germinate [50% germinated (days)] was calculated from the curves. Seedling growth time [2 cm root length (days)] was calculated by subtracting the 50% germination time from the time for the roots to grow to 2 cm for each seedling.

## Results

### Generation of transgenic plants overexpressing *BASS2* driven by the *glycinin-1* seed-specific promoter

**Figure 2:**
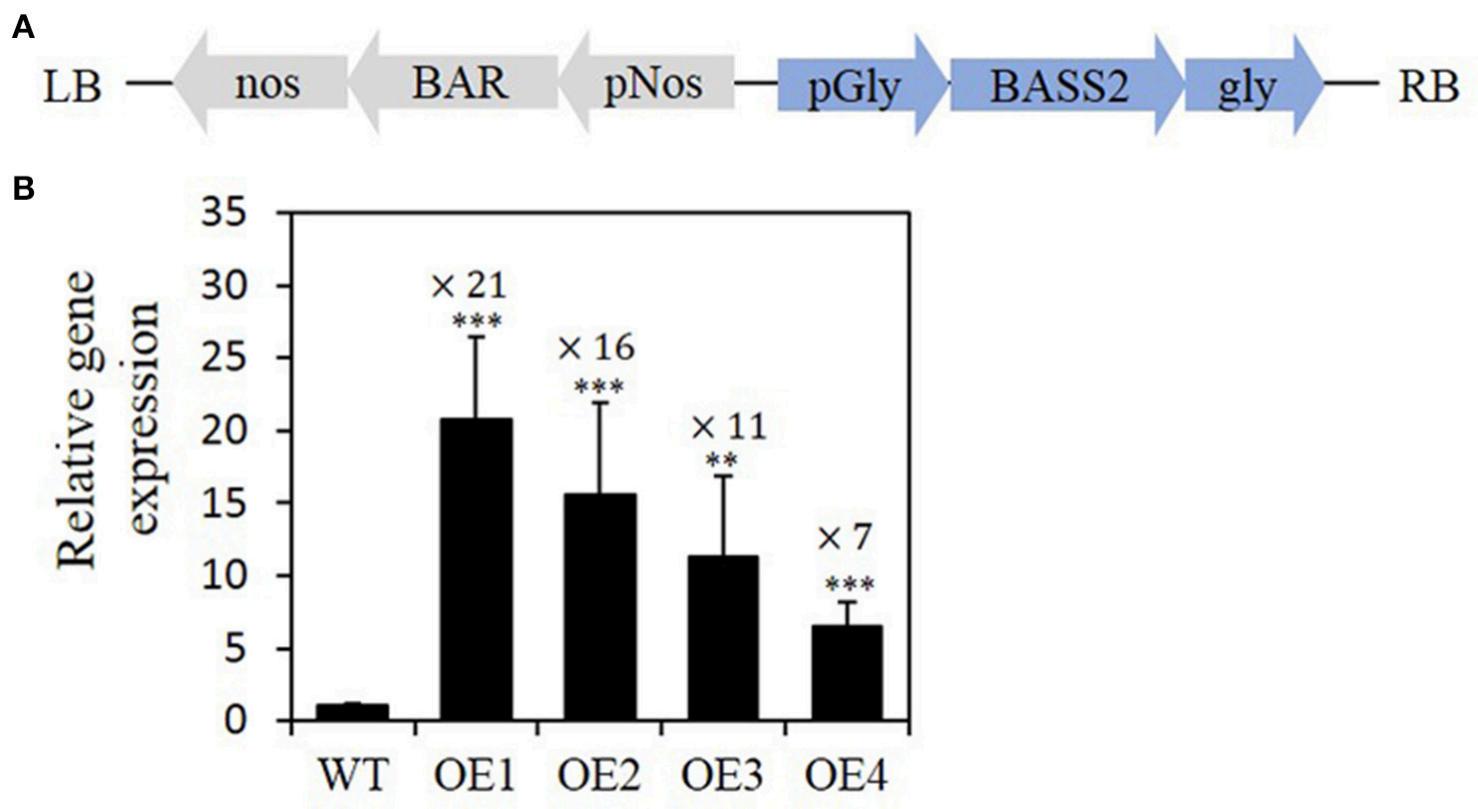
**Expression levels of the pyruvate transporter (***BASS2***) in the developing siliques of transgenic plants overexpressing ***BASS2***. (A)** Schematic representation of the T-DNA of the binary vector used to express *BASS2* under the control of the seed-specific soybean *glycinin-1* promoter. **(B)** Relative levels of *BASS2* expression. RNA was extracted from T2 developing siliques (12–14 DAF) of four independent *BASS2*-overexpressing lines (OE1, OE2, OE3, and OE4) and the wild type (WT). *BASS2* transcript levels were determined by real-time quantitative RT-PCR, normalized to transcript levels of the control gene UBQ11, and presented relative to values of the WT, which were set to 1. Error bars depict standard error (±SE; *n* = 3). Asterisks indicate significant difference from the WT (*N* = 3, 6 ≤ *n* ≤ 15, ^**^P < 0.01, ^***^P < 0.001), as determined using Student's *t*-test. Values above the columns indicate fold changes when compared with WT. pGly, soybean *glycinin-1* promoter; *BASS2*, bile-acid sodium symporter 2; gly, *glycinin-1* terminator; pNos, nopaline synthase promoter; BAR, BASTA resistance gene; nos, nopaline synthase terminator sequence; LB, left border; RB, right border; DAF, day after flowering.

Constitutive overexpression of a gene that increases seed oil content often results in plants with decreased height and seed yield (Li et al., 2013; Guo et al., 2014). To overcome such limitations, we overexpressed the *BASS2* coding sequence in *Arabidopsis* under the control of the seed-specific promoter of soybean (*Glycine max*) *glycinin-1* (resulting in transgenic lines OE1–OE4; Figure 2A). *Glycinin-1* encodes one of the major seed storage proteins in soybean and is expressed during the mid to late stages of seed development (Nielsen et al., 1989; Iida et al., 1995). We initially selected 65 transgenic plants on antibiotics-containing selection medium, and then measured their seed size using Image J, as explained in Materials and Methods. An increase in seed size was observed in 49 lines of the transgenic plants (75%). Six independent lines of the transgenic seeds were sown to obtain the next generation of plants.

We next performed quantitative RT-PCR analysis to confirm that *BASS2* was overexpressed in the seeds of the T2 generation. We evaluated the expression of *BASS2* in the developing siliques 12–14 days after flowering (DAF), when the biosynthesis and accumulation of storage lipids increase drastically (Ruuska et al., 2002). We then chose four lines that showed a range of *BASS2* transcript levels in siliques containing developing seeds. *BASS2* gene expression levels in the developing siliques of OE1, OE2, OE3, and OE4 plant lines were found to be 21-, 16-, 11-, and 7-fold higher than that in the WT at a similar developmental stage, respectively (Figure 2B). The data presented below are based on the seeds produced by the T2 generation.

### Seed-specific overexpression of *BASS2* increases the seed oil content

**Figure 3:**
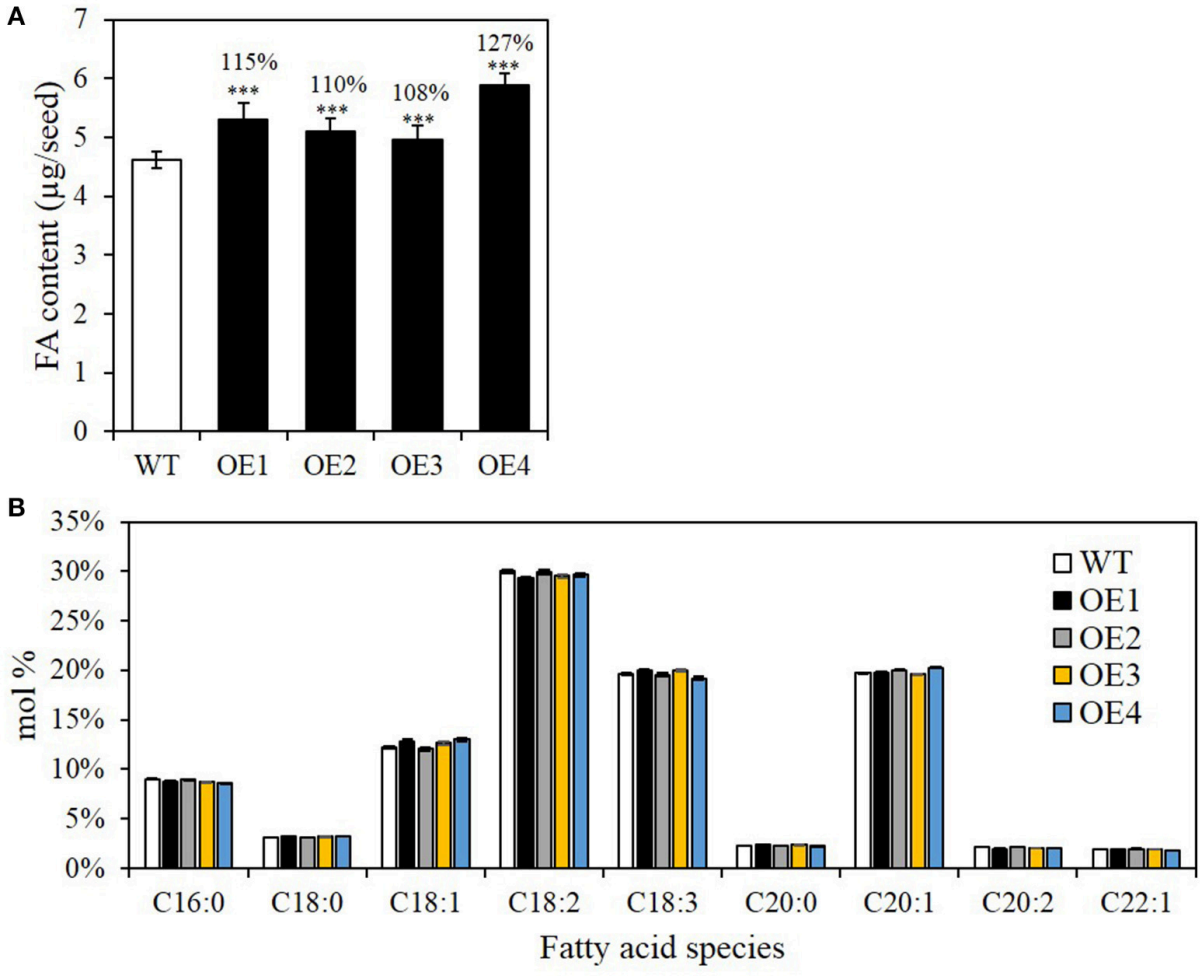
**Seed oil contents and fatty acid (FA) composition in seed-specific ***BASS2***-overexpressing (OEs) seeds. (A)** The oil contents of seeds produced by the T3 WT and OE lines. **(B)** FA composition (mol%) of seeds of WT and OE lines. Error bars depict standard error (±SE). Asterisks indicate significant difference from the WT (*N* = 3, 24 ≤ *n* ≤ 90, ^***^P < 0.001), as determined using Student's *t*-test. Values above the columns indicate FA content as a percentage of that in WT seeds.

We firstly examined whether seed-specific *BASS2* overexpression increased the seed oil content by measuring the amount of total fatty acid methyl esters (FAMEs), which reflect changes in seed oil content, because >94% of fatty acids in seeds are stored in the form of TAG (Li et al., 2006). The total seed oil content in OE1, OE2, OE3, and OE4 was significantly increased by 15, 10, 8, and 27%, respectively, compared with those of WT (Figure 3A). The fatty acid composition of seeds of the OE lines was indistinguishable from that of the WT (Figure 3B), suggesting that *BASS2* overexpression did not affect fatty acid desaturations or elongations. C20:1 FA is present almost exclusively in seed TAG, and is often used as a marker of TAG content (Lemieux et al., 1990). C20:1 FA levels in OE1, OE2, OE3, and OE4 seeds were 32, 13, 10, and 37% higher than in WT seeds, respectively. There was also a net increase in quantity of almost all FAs (Supplementary Figure 2). Thus, the seed-specific *BASS2* overexpression increased general fatty acid biosynthesis and thereby TAG biosynthesis, but did not affect fatty acid modifications.

### Seed-specific *BASS2* overexpression increases seed biomass, but does not alter the protein and carbohydrate content

**Figure 4:**
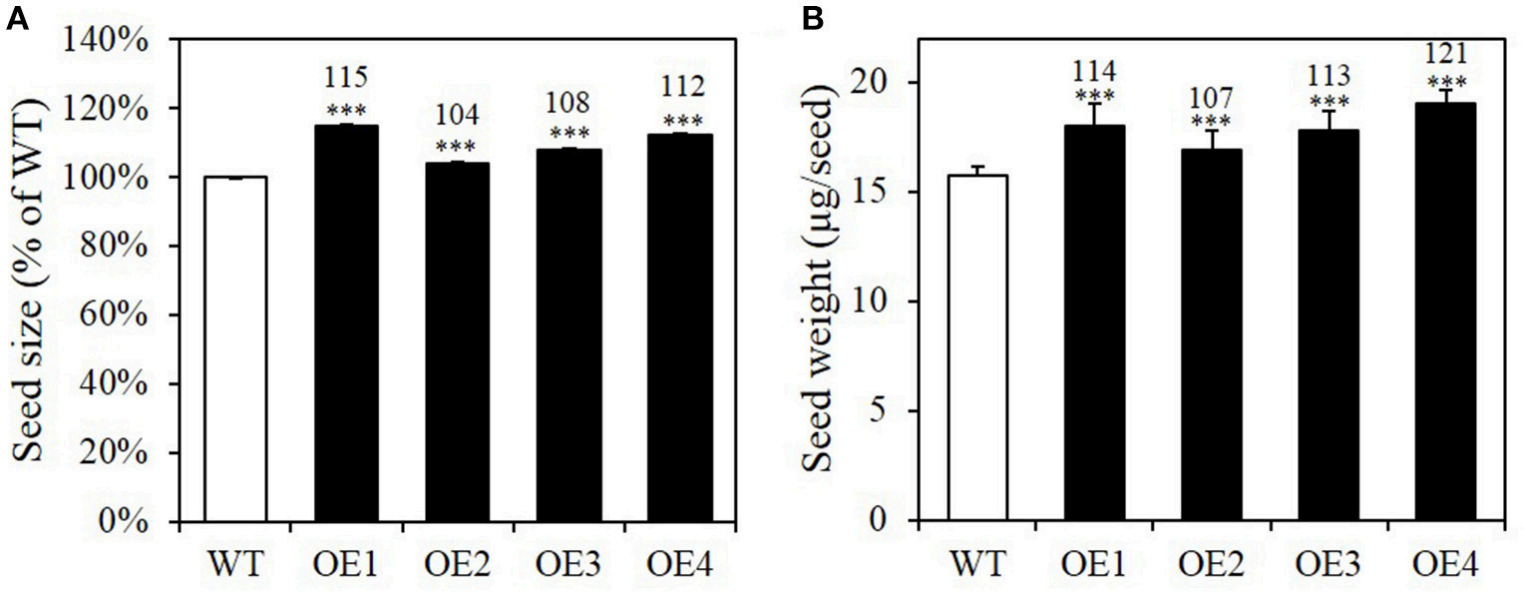
***BASS2*****-overexpressing plants produce larger and heavier seeds than the WT. (A)** Seed size of wild-type (WT) and *BASS2*-overexpressing lines (OE1, OE2, OE3, and OE4). The T3 seeds of *BASS2*-overexpressing lines were photographed and the mean values of the cross sectional area were compared to that of WT. Error bars depict standard error (±SE; *n* = 3). Asterisks indicate significant difference from the WT (1092 ≤ *n* ≤ 4308, ^***^P < 0.001), as determined using Student's *t*-test. Values above the columns indicate seed size as a percentage of that of the WT. **(B)** Seed weight of WT and OEs. For each replicate, 300 seeds of the WT and *BASS2*-overexpressing lines (OEs) were collected and weighed. Seed weight was positively correlated with seed size. Error bars depict standard error (±SE). Asterisks indicate significant difference from the wild-type (*N* = 3, 8 ≤ *n* ≤ 30, ^***^P < 0.001) as determined using Student's *t*-test. Values above the columns indicate seed weight as a percentage of that of the WT.

Pyruvate is an intermediate not only in the biosynthesis of oil, but also of branched chain amino acid (BCAA) and terpenoid biosynthesis in the plastid. Besides oil, the other major components of *Arabidopsis* seeds are proteins and carbohydrates (Li-Beisson et al., 2010). We thus first investigated whether *BASS2* overexpression induced an increase in seed biomass, by measuring the size and weight of *BASS2* overexpressing seeds (Figure 4, Supplementary Figure 1). T2 seeds of the *BASS2* overexpression lines were imaged and the mean values of the cross sectional area were compared to those of the WT. We found that the OE1, OE2, OE3, and OE4 lines produced seeds that were 132, 112, 112, and 121% larger than those of WT (Supplementary Figure 1). The increase in seed size was confirmed in T3 lines; the seeds of OE1, OE2, OE3, and OE4 lines of the T3 generation were 115, 104, 108, and 112% the size of WT seeds, respectively (Figure 4A). Consistent with the increase in seed size, *BASS2*-overexpressing seeds were significantly heavier than those of the WT (Figure 4B). The average seed weights measured from 300 seeds of the *BASS2* overexpression lines were 7–21% larger than those of the WT.

**Figure 5:**
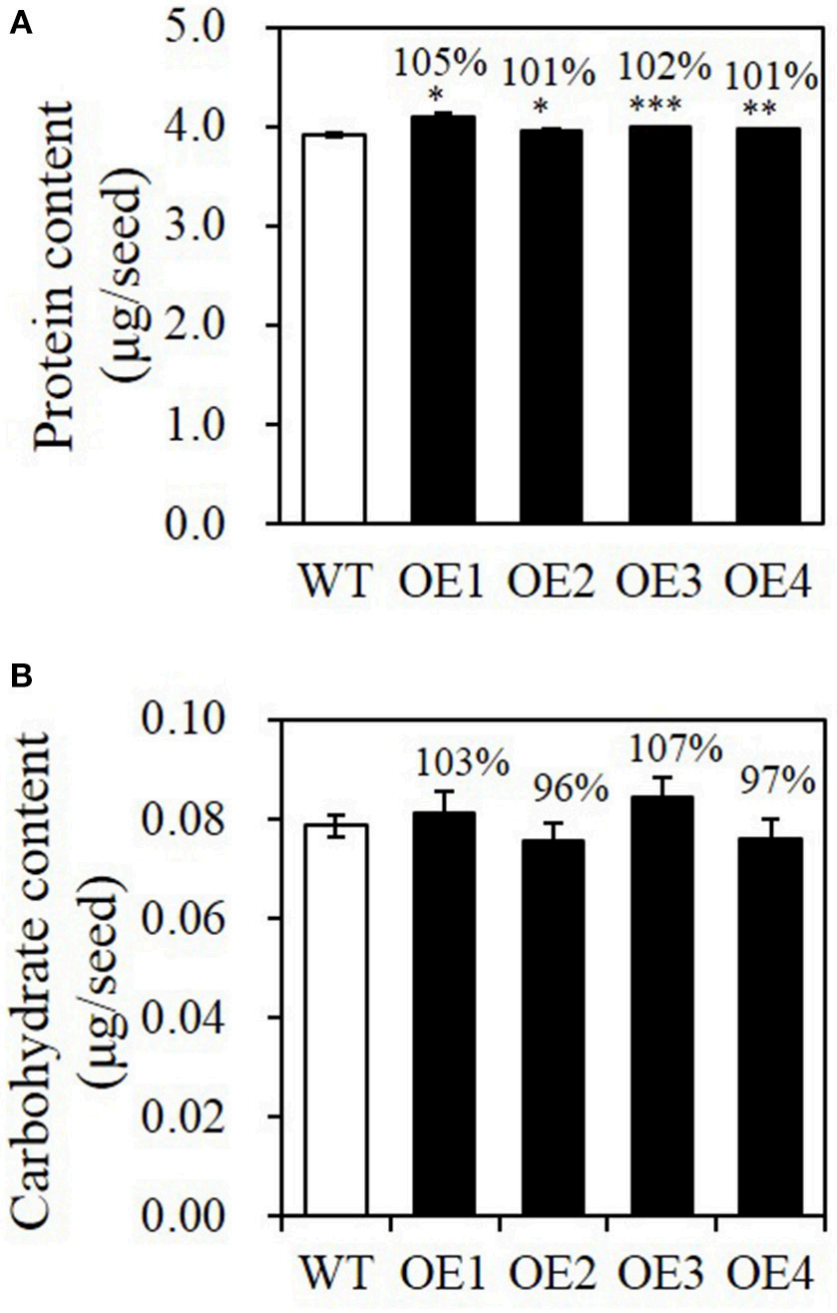
**Protein and carbohydrate contents of WT and ***BASS2***-overexpressing (OEs) seeds. (A,B)** Protein and carbohydrate contents in T3 seeds of WT and *BASS2*-overexpressing lines. Carbohydrate contents were measured as the sum of sucrose and starch extracts. Values are the mean contents of each metabolite ±SE as a percentage of the corresponding WT value, which was set to 100%. Asterisks indicate significant difference from the wild-type (^*^P < 0.05, ^**^P < 0.01, ^***^P < 0.001), as determined using Student's t-test. **(A)** N = 3, 42 ≤ *n* ≤90, for OE2, OE3, and OE4. N = 1, *n* = 3 for OE1. **(B)** N = 3, 6 ≤ *n* ≤ 29 for all samples.

Because seed biomass was increased in the *BASS2* overexpression lines, we determined whether other storage substances increased accordingly in the OE lines. As shown in Figure 5A, the total protein contents were similar among all lines tested, i.e., about 4 μg per seed, although the levels were slightly higher in the OEs than in the WT. Carbohydrate content, i.e., the sum of starch and sucrose contents, was much lower than those of other storage compounds, and varied among *BASS2*-OE lines, with no statistically significant difference between the seeds of different genotypes (Figure 5B).

### Seed vigor traits of seed-specific *BASS2* overexpression lines are comparable to those of the WT

**Figure 6:**
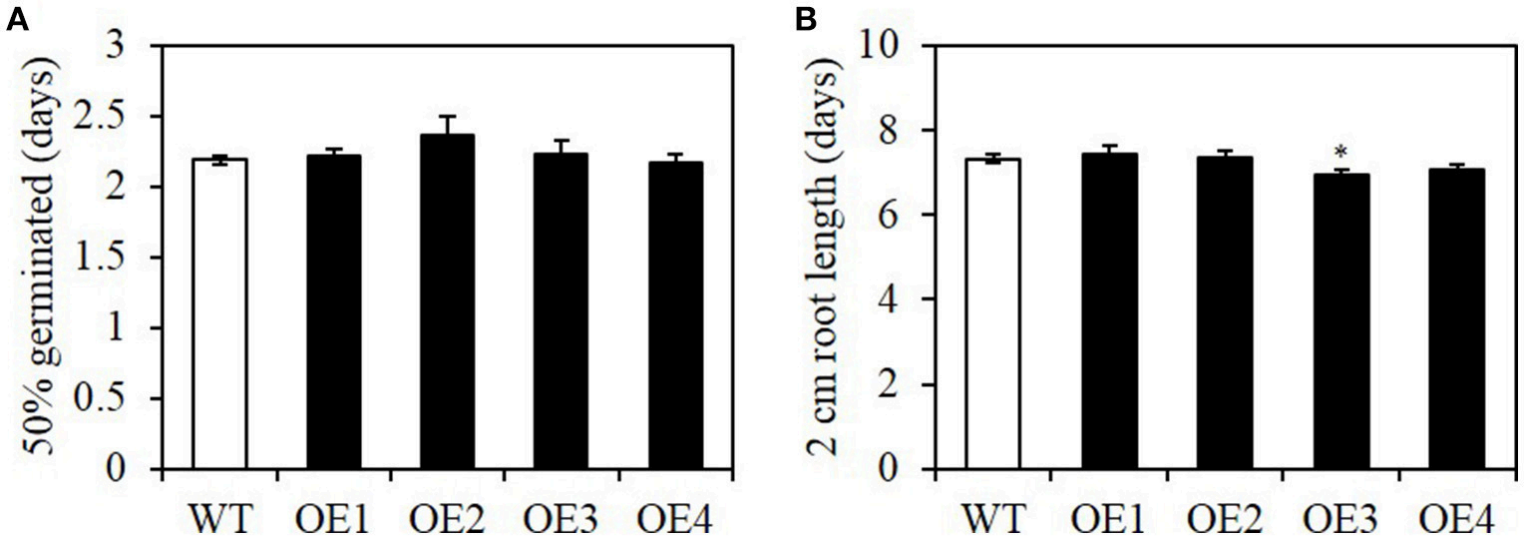
**Germination rate and early seedling growth rate of seed-specific ***BASS2***-overexpressing lines (OEs)**. The time required for 50% of the seeds to germinate **(A)** and the time for seedlings to grow roots to 2 cm **(B)** were scored. Values are means ±SE. *N* = 2, 29 ≤ *n* ≤ 71, Student's *t*-test (^*^P < 0.05).

Seed vigor, including rapid uniform germination and seedling growth, is an important agronomic trait (Finch-Savage et al., 2010). To determine whether seed-specific overexpression causes any negative impact on seed vigor, we measured the germination rate and seedling growth rate of OE lines. The average time required for 50% of the seeds to germinate was not significantly different between the WT and OE lines (Figure 6A). The initial seedling growth rate, measured as the period between germination and the seedling root length reaching 2 cm, was also similar (Figure 6B). Therefore, the seed-specific overexpression of *BASS2* did not affect germination or initial seedling establishment.

### The *BASS2* overexpressing lines produce larger seeds without compromising seed number

**Figure 7:**
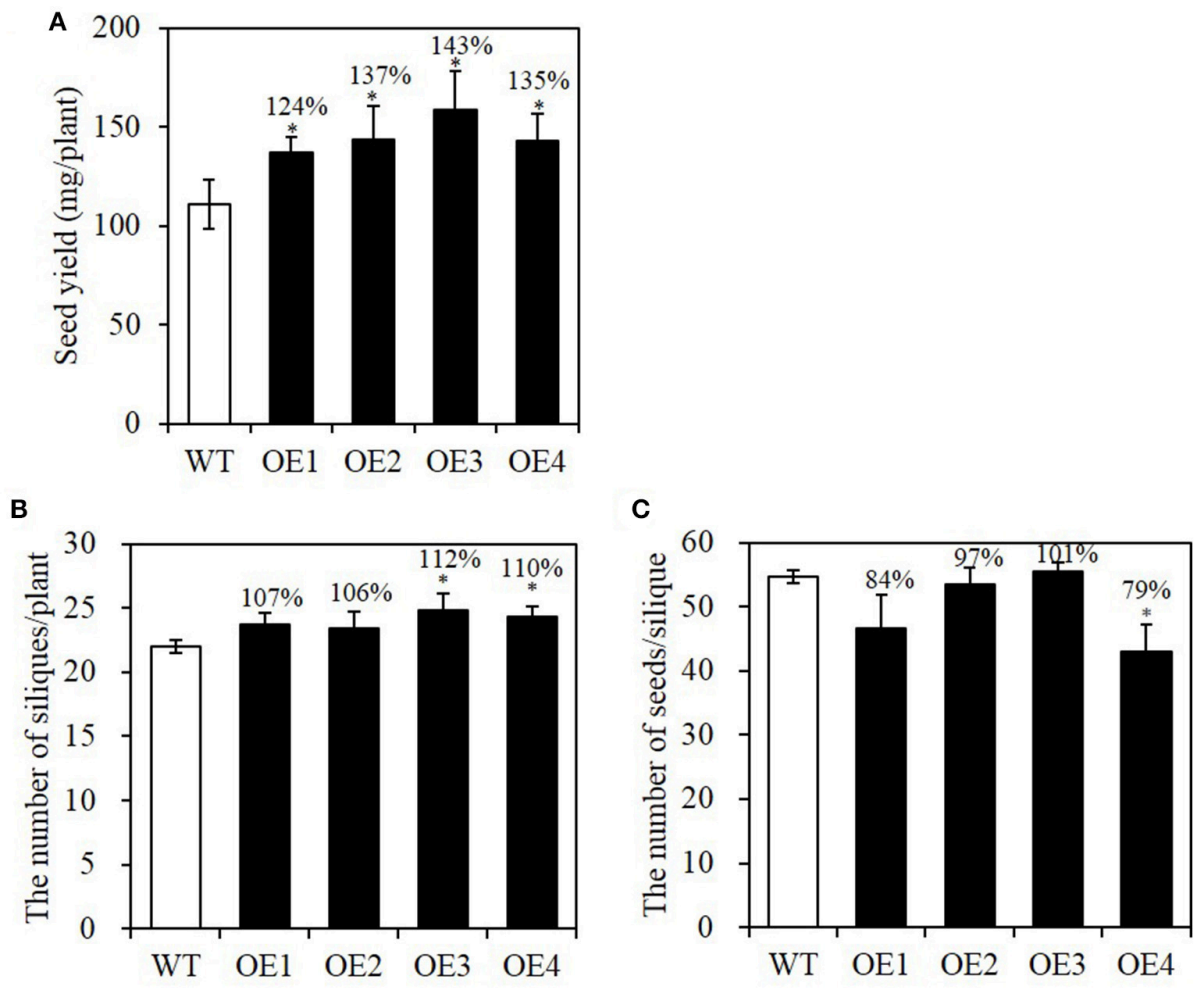
**Seed yield of WT and seed-specific ***BASS2***-overexpressing (OEs) plants. (A)** Seed yield of WT and OEs. Seed yield of plants was measured as the total seed weight from a plant (*N* = 2, *n* = 7, 8). **(B)** Silique number of the main stem of WT and OE plants. After inflorescence meristem growth of the main stem had ceased, the number of siliques on the main stem was counted (*N* = 6, 10 ≤ *n* ≤ 30). **(C)** Seed number per silique. Developing siliques were sampled, and the seed number in the siliques was counted under a dissecting microscope (*N* = 3,6 ≤ *n* ≤ 14). Error bars indicate standard error (SE). Asterisks indicate significant difference from the WT (^*^P < 0.05), as determined using Student's *t*-test. Values above the columns indicate seed yield, the number of siliques per plant or the number of seeds per silique as a percentage of the corresponding WT value.

Total seed yield is an important factor contributing to net oil production in an agricultural field. Because increased seed size is often offset by decreased seed number in a silique, we examined seed yield in each OE plant as well as silique number and seed number per silique. First, seed-specific overexpression plants of *BASS2* were grown and the seeds from those plants were harvested to compare total seed yield. The seed yield (mg per plant) was 24–43% higher in the OE lines than in the WT (Figure 7A).

To test whether the increased seed yield was due to an increase in silique number or seed number per silique, we counted the number of siliques on the main stem and the seed number per silique when flowering ceased and the siliques began to turn yellow. As shown in Figure 7B, the silique number on the main stem was comparable to that of WT. Only OE3 and OE4 lines showed slight increases, of about 10%, compared with the WT. The seed number per silique did not differ significantly from the WT, except in OE4 (Figure 7C). The total seed number of OE plants, estimated from these values, was indistinguishable from that of WT; the total seed numbers among different OE lines varied between 87 and 113% of the WT value, and no significant difference was observed. Therefore, we concluded that silique number or seed number per silique did not change in the seed-specific overexpressing lines of *BASS2*. This implies that the total yield increase observed in the *BASS2*-overexpressing lines was mainly due to an increase in individual seed weight (Figure 4B).

## Conclusion and discussion

In this study, we show that increasing pyruvate flux into the plastids of developing seeds can increase seed lipid content. Four independent OE lines showed increased *BASS2* expression levels in developing siliques (Figure 2B) and produced larger seeds than those of the WT in both the T2 and T3 generations (Figure 4A, Supplementary Figure 1). We also showed that the seed oil content was greater in the OE lines than in the WT (Figure 3A, Table 1), but that the protein and carbohydrate contents of seeds were comparable among the OE and WT lines (Figures 5A,B). These results suggest that the increase in seed weight in the OE lines was mainly due to the increased seed oil content, and not due to changes in other seed storage compounds (Table 1).

**Table 1:** **Lipid, protein, and carbohydrate contents of WT and ***BASS2***-overexpressing (OE) seeds**.

**Parameter**	**WT**	**OE1**	**OE2**	**OE3**	**OE4**
Seed weight, μg/seed	15.74 ± 0.43	18.00 ± 1.02^***^	16.89 ± 0.92^***^	17.79 ± 0.88^***^	19.01 ± 0.61^***^
Lipid weight, μg/seed	4.62 ± 0.14	5.31 ± 0.28^***^	5.09 ± 0.23^***^	4.97 ± 0.23^***^	5.88 ± 0.20^***^
Protein weight, μg/seed	3.92 ± 0.01	4.10 ± 0.04^*^	3.96 ± 0.02^*^	3.99 ± 0.01^***^	3.97 ± 0.01^**^
Carbohydrate weight, μg/seed	0.079 ± 0.002	0.081 ± 0.004	0.075 ± 0.004	0.084 ± 0.004	0.076 ± 0.004
Total reserves, μg/seed	8.62	9.49	9.13	9.04	9.93
ΔSeed weight (OE-WT), μg/seed		2.26	1.15	2.05	3.27
ΔSeed reserve weight (OE-WT), μ g/seed		**0.87**	**0.51**	**0.43**	**1.31**
ΔLipid weight (OE-WT), μg/seed		**0.69**	**0.47**	**0.35**	**1.26**
Lipid, % of dry weight	28.65 ± 0.92	29.19 ± 1.70	29.67 ± 0.94	27.57 ± 1.45	30.59 ± 1.07
Protein, % of dry weight	25.45 ± 0.73	21.29 ± 1.33^*^	24.41 ± 1.29	23.13 ± 1.10^*^	21.17 ± 0.71^***^
Carbohydrate, % of dry weight	0.51 ± 0.02	0.46 ± 0.04	0.45 ± 0.02^*^	0.48 ± 0.03	0.40 ± 0.02^***^

*Values are averages ± SEs from three replicates. Numbers in red indicate values that are significantly different from those of the WT at P < 0.001. Note that the increase in seed reserve weight is related mainly to an increase in lipid weight (numbers in bold)*.

* P < 0.05;

** P < 0.01;

*** *P < 0.001, Student's t-test*.

The additional increase in seed weight may have been due the increase in cell wall and seed coat compounds, which would have accompanied the increase in seed size. Most importantly, the changes in seed reserves of the four OE lines (0.87, 0.51, 0.43, and 1.31 μg/seed, respectively) corresponded closely with the change in their lipid weight (0.69, 0.47, 0.35, and 1.26 μg/seed). The lipid content as a percentage of the total seed weight (% of dry weight) was similar in the OE and WT seeds. Moreover, *BASS2* overexpression resulted in non-selective increases in all fatty acid species including C20:1 (Figure 3B, Supplementary Figure 2), suggesting an overall increase in cellular fatty acid biosynthesis, i.e., in both plastids (*de novo*) and the endoplasmic reticulum (ER) (elongation). This is consistent with the idea that partitioning more pyruvate into the plastids may provide more acetyl-CoA for synthesis of all fatty acids.

Constitutive overexpression of genes often causes a tradeoff between seed size/oil content and seed number. Our data show that this problem did not occur when a seed-specific *glycinin-1* promoter of soybean was used; in OE lines, seed number per silique was similar to that of the WT (Figure 7C) and the total seed yield (mg) of the plant increased (Figure 7A), suggesting that seed oil production can be improved without altering the total seed number produced per plant. The increased total seed yield in OE lines was due mainly to the increase in individual seed weight. Moreover, *BASS2* overexpression did not alter physiological processes of the plants, such as seed germination and seedling growth were not compromised in the OEs (Figure 6). This is of economic value in real-world situations.

In addition to fatty acid biosynthesis, pyruvate can be used in plastids for many biosynthetic pathways, including the biosynthesis of terpenoids and BCAAs (Schulze-Siebert et al., 1984; Hemmerlin et al., 2003; Schwender et al., 2004). However, it seems unlikely that pyruvate is used for the terpenoid biosynthesis pathway in the seeds of the overexpressors, since terpenoids are not a major constituent of *Arabidopsis* seeds. Neither are BCAAs a major constituent of seed weight that could explain the increase in seed weight we observed in *BASS2* overexpressors; BCAAs are only minor components of *Arabidopsis* seeds (0.67 nmol/mg; <0.1 μg/mg dry seeds; Angelovici et al., 2013). The storage protein content, which accounts for about 30% of the *Arabidopsis* dry seed weight, did not differ much between the OE and WT lines (Figure 5A). Thus, the additional pyruvate compartmentalized into plastids by overexpressed *BASS2* seems to have been used mainly for lipid biosynthesis. This may be, at least in part, because the *glycinin-1* promoter activity is highest during the maturation phase of seed development (Li et al., 2015), when lipid biosynthesis sharply increases in *Arabidopsis* seeds (Nielsen et al., 1989; Baud et al., 2002; Ruuska et al., 2002).

Taken together, our study demonstrates that overexpressing a plastidial pyruvate transporter driven by a seed-specific promoter is a useful approach for increasing the oil content without impacting the deposition of other storage materials in seeds. Despite their obvious importance in transporting metabolites across distinct subcellular compartments housing lipid biosynthesis, transporter genes have only recently been identified and explored as a strategy to increase seed oil yield. Overexpression of *AtABCA9*, which is localized to the ER, enhanced TAG content by up to 40%, most likely by facilitating the transport of fatty acids to the site of TAG biosynthesis (Kim et al., 2013). Thus, genetic engineering employing organellar transporters can be used to increase the flux between organelles, resulting in increased seed oil yield. This study provides an additional element (i.e., *BASS2*) that can be manipulated to further increase oil content using a gene stacking approach. For example, overexpression of *BASS2* and other factors such as *AtWRI1* or *AtABCA9*, or overexpression of *BASS2* in lines in which lipid catabolism is shut down might result in lines with increased oil contents. Such approaches might be applicable to oil seed crops, such as rapeseed, flax, and sunflower.

## Author contributions

EL, YL-B, and YL designed the research; EL and MO performed the experiments; JH, YL-B, and IN analyzed the data; EL, YL-B, and YL wrote the manuscript.

## Funding

This research was supported by the Advanced Biomass R&D Center (ABC) of Global Frontier Project funded by the Ministry of Science, ICT and Future Planning (ABC- 2015M3A6A2065746) of the Republic of Korea awarded to YL, by JSPS KAKENHI Grant Number 24570040 to IN, and by MUsCA grant to YL-B.

### Conflict of interest statement

EL, MO, and YL have filed a patent PCT/KR2015/009411 entitled, “Composition for increasing seed size and content of storage lipid in seed, comprising BASS2 protein or coding gene thereof.” The other authors declare that the research was conducted in the absence of any commercial or financial relationships that could be construed as a potential conflict of interest.
