# Supplementary material for: Seed-Specific Overexpression of the Pyruvate Transporter BASS2 Increases Oil Content in Arabidopsis Seeds
Source: Front Plant Sci. 2017 Feb 20;8:194. doi: 10.3389/fpls.2017.00194 (PMC5316546; doi:10.3389/fpls.2017.00194)
Supplement: Supplementary file 1 [file Image1.PDF]

Supplementary Material

Seed-specific overexpression of the pyruvate transporter BASS2 increases oil content in *Arabidopsis* seeds

Eun-Jung Lee[1], Minwoo Oh[1], Jae-Ung Hwang[1], Yonghua Li-Beisson[2], Ikuo Nishida[3], Youngsook Lee[1*]

[*]**Correspondence** :

Youngsook Lee

ylee@postech.ac.kr

Supplementary Figures

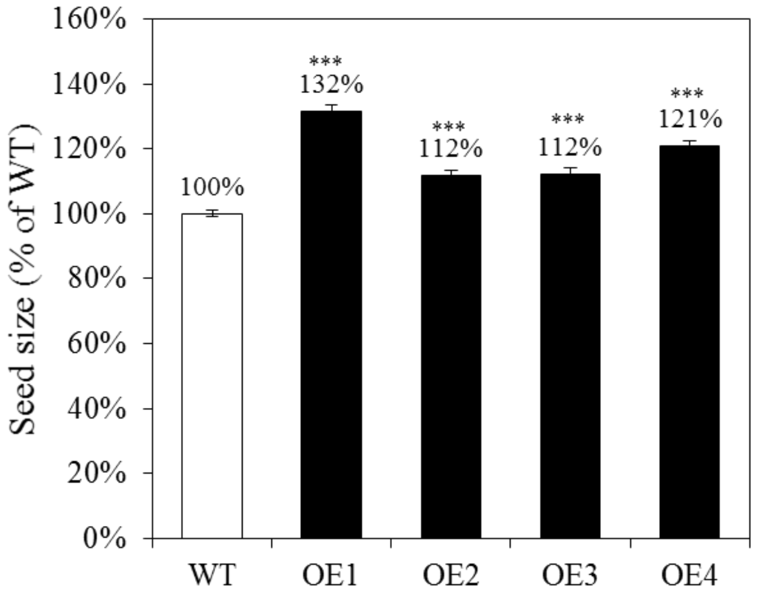

Supplementary Figure 1. The seed size of T2 lines in seed-specific *BASS2*-overexpressing lines (OEs)

The seed size was measured using Image J software. The increase in seed size exhibited a similar tendency as in the T3 lines (Figure 4A), although the percentage of seed size increase in the T2 lines was higher than that in T3. Values are means ±SE. N= 1,57 $\leq n \leq$ 68, Student's *t*-test (**P* < 0.05, ***P* < 0.01 ****P* < 0.001)

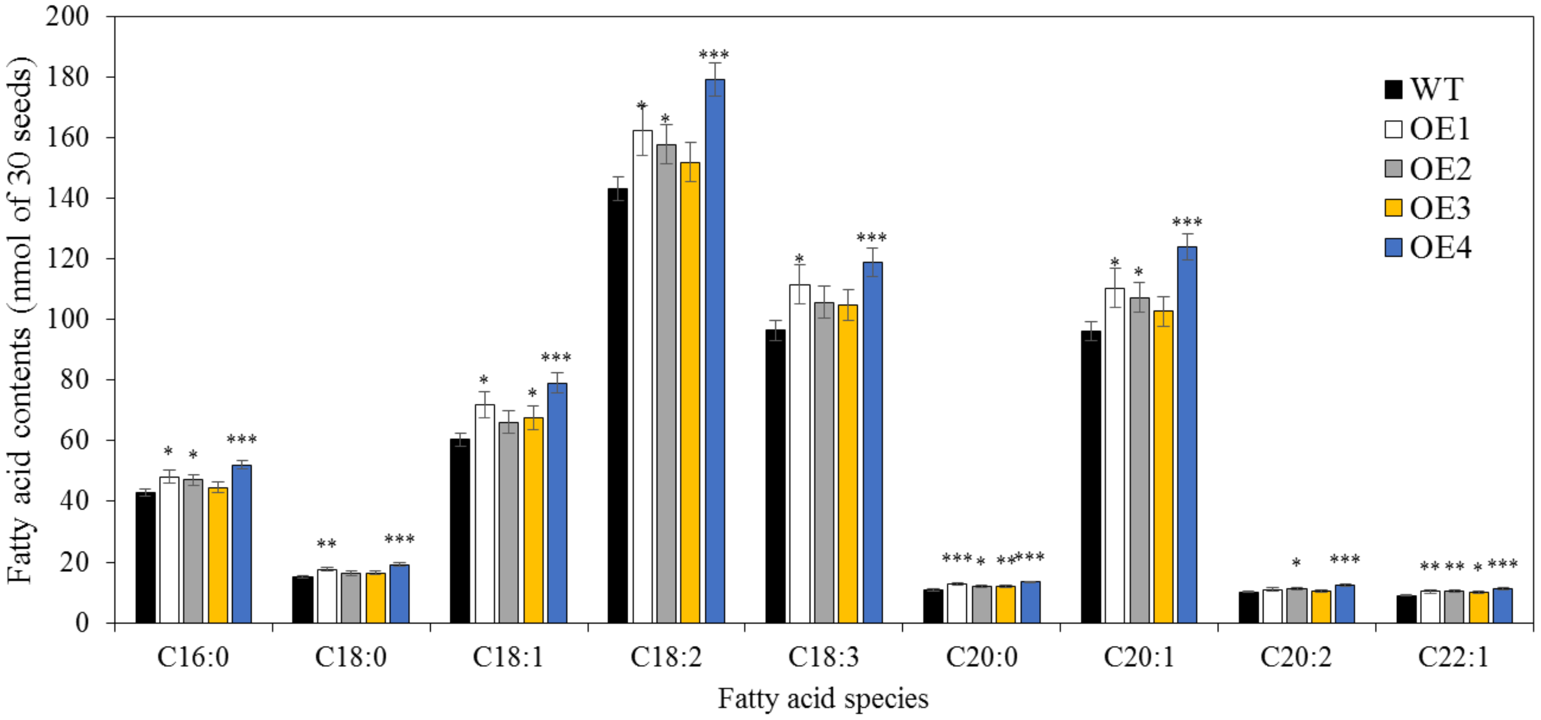

Supplementary Figure 2. Fatty acid content (in absolute values) in seed-specific *BASS2*-overexpressing (OEs) seeds.

Fatty acid methyl esters (FAMEs) from seeds of WT and OEs were quantified using gas chromatography-mass spectrometry (GC-MS). Error bars depict standard error (±SE). Asterisks indicate significant difference from the wild-type (N=3, 24≤*n*≤90, *P < 0.05, **P < 0.01, ***P < 0.001).
